# Fine‐scale substrate heterogeneity in green roof plant communities: The constraint of size

**DOI:** 10.1002/ece3.5517

**Published:** 2019-09-30

**Authors:** Amiel Vasl, Bracha Y. Schindler, Gyongyver J. Kadas, Leon Blaustein

**Affiliations:** ^1^ Kadas Green Roofs Ecology Research Center Institute of Evolution and Department of Evolutionary and Environmental Biology Faculty of Natural Sciences University of Haifa Haifa Israel; ^2^ Environmental Research Group Sustainability Research Institute University of East London London UK

**Keywords:** neutral theory, niche theory, plant community assemblage, plant–soil interactions

## Abstract

Heterogeneity–diversity relationship (HDR) is commonly shown to be positive in accordance with classic niche processes. However, recent soil‐based studies have often found neutral and even negative HDRs. Some of the suggested reasons for this discrepancy include the lack of resemblance between manipulated substrate and natural settings, the treated areas not being large enough to contain species' root span, and finally limited‐sized plots may not sustain focal species’ populations over time. Vegetated green roofs are a growing phenomenon in many cities that could be an ideal testing ground for this problem. Recent studies have focused on the ability of these roofs to sustain stable and diverse plant communities and substrate heterogeneity that would increase niches on the roof has been proposed as a method to attain this goal. We constructed an experimental design using green roof experimental modules (4 m^2^) where we manipulated mineral and organic substrate component heterogeneity in different subplots (0.25 m^2^) within the experimental module while maintaining the total sum of mineral and organic components. A local annual plant community was seeded in the modules and monitored over three growing seasons. We found that plant diversity and biomass were not affected by experimentally created substrate heterogeneity. In addition, we found that different treatments, as well as specific subplot substrates, had an effect on plant community assemblages during the first year but not during the second and third years. Substrate heterogeneity levels were mostly unchanged over time. The inability to retain plant community composition over the years despite the maintenance of substrate differences supports the hypothesis that maintenance of diversity is constrained at these spatial scales by unfavorable dispersal and increased stochastic events as opposed to predictions of classic niche processes.

## Graphical abstract

The unique negative/neutral heterogeneity–diversity relationships that are found in plant‐soil study are displayed in our green roof study. Our findings allow to examine some of the existing theory and suggest towards the population viability theory.

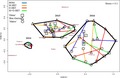

## 1 INTRODUCTION

One of the longest standing challenges in the field of ecology is explaining the mechanisms that sustain species richness over time and space. Spatial heterogeneity of resources and environmental conditions was suggested to increase niches which would, in turn, support the maintenance of a variety of species (Chesson, 2000; MacArthur & Levins, 1964). Plants were previously used to show that the maintenance of a diverse plant community is a direct result of fine‐scale heterogeneity where different plant species are supported by different patches (Whittaker, 1965).

Accumulating evidence for contradicting hypotheses resulted in the publication of the neutral theory (Hubbell, 2001) that successfully predicted observed patterns while completely ignoring resource heterogeneity. Although these contradicting theories were generally reconciled into a “niche–neutral continuum” (Leibold & McPeek, 2006; Matthews & Whittaker, 2014), the underlying insight was that the seemingly obvious heterogeneity–diversity relationship (i.e., HDR) was no longer indisputable.

This shaking of the niche theory may have given rise to the emergence of several studies that have challenged the generality of positive HDR especially in soil heterogeneity and even suggested negative HDRs (Gazol et al., 2013; Lundholm, 2009; Tamme, Hiiesalu, Laanisto, Szava‐Kovats, & Pärtel, 2010). Experimental studies that put this theory to test only rarely found a positive HDR for soil heterogeneity (Williams & Houseman, 2014). A large‐scale meta‐analysis was performed (Stein, Gerstner, & Kreft, 2014) and showed a significantly positive HDR effect across taxa, biomes, and spatial scales which could have potentially refuted the negative HDR studies. However, the meta‐analysis only included large‐scale (>10 km^2^) observational studies while the contradictory results were attained in experimentally manipulated fine‐scale studies.

Some have tried linking this discrepancy to the effect of patch size. A meta‐analysis performed on soil manipulations studies (Tamme et al., 2010) claimed that experimental studies' negative HDR was limited by fine‐scale patch size where fine‐scaled heterogeneity supported lower diversity. This is also supported by the strong positive effect of patch size found in the meta‐analysis performed on observational soil studies (Tamme et al., 2010) and, in general, HDR studies (Stein et al., 2014). Since experimental studies are inherently limited in their dimensions, it can be suggested that the manipulated patch size is innately limited by experimental dimensions due to physical restrictions which may explain the scarcity of positive HDR effects.

The attempt to reconcile negative HDRs in experimentally manipulated studies with the general positive perceived trend received three different potential hypotheses that were suggested or tested: lack of realism is embedded in the method of man‐made heterogeneity, patch size effect on individuals, and patch size effect on populations.

Hypothesis 1: Realism in the method of creation of heterogeneity.

It has been claimed that a lack of realism is inherent in most methods of heterogeneity manipulation, especially with nutrient manipulations (Williams & Houseman, 2014). The manipulated substrates may not mimic natural soil, and nutrients that are artificially added may disturb plant–soil microbe interactions or, in certain cases where highly mobile forms of nitrogen are used, give preference to nitrophilic species that are able to capitalize on the resources more easily, which masks the heterogeneity effect.

Hypothesis 2: Patch size has effect on individuals.

Treated patch size within experimental modules has been targeted for some time as a potential challenge in studies of this kind; treated areas that are smaller than the root span of certain species are functionally invisible to those species (Hutchings, John, & Wijesinghe, 2003). However, when all species have similar root spans that are larger than treated patches, the heterogeneity effect is predicted to be neutral. When some species' root spans are smaller and some are larger than patch size, species with larger root spans have a foraging advantage over species with smaller root spans and increase their fitness which could potentially reduce diversity (Rajaniemi, 2011; Tamme, Gazol, Price, Hiiesalu, & Pärtel, 2016).

Hypothesis 3: Patch size has effect on populations.

Theoretical models designed to improve our understanding of community dynamics within heterogeneous surroundings found support for the negative HDR (Kadmon & Allouche, 2007; Palmer, 1992; Smith & Lundholm, 2012). This is explained by the increased stochasticity caused by habitat heterogeneity which affects plant populations. Reducing the absolute patch area results in smaller populations in each of the patches which in turn increase the chances of stochastic events occurring within them. An important role was also assigned to dispersal mechanisms—smaller patches would increase the percentage of propagules dispersed from the patches into unsuitable habitats due to the fact that patch perimeter would be closer to the plant and would also reduce the incoming propagules from the regional species pool (Kadmon & Allouche, 2007). At reduced patch sizes, increased heterogeneity has a better chance of causing a negative HDR.

In this experiment, we wish to put two of these hypotheses (2 and 3) to test. The construction of large experimental modules (=units) with large enough subplots (=patches) to sustain distinct plant populations and communities and manipulating mineral substrate components alongside observation and sampling over several years will allow us to examine the first and third hypotheses more closely. HDR as well as comparing community compositions between treated modules and subplots within modules could potentially shed light on the processes taking place. While substrate heterogeneity was predicted to increase plant diversity, we did not expect that it would increase plant biomass.

The increasingly common green roof studies may serve as an ideal testing ground for questions of this type. Green roofs are a widespread urban phenomenon where a vegetative layer is placed on roofs. The majority of green roofs are lightweight and often planted with a small array of plant species that entail minimal maintenance (Oberndorfer et al., 2007). While green roofs were originally designed to mitigate stormwater runoff and enhance buildings' thermal insulation, their potential ecological benefits such as increasing biodiversity have been receiving more focus in past years (Blaustein, Kadas, & Gurevitch, 2016; Lundholm & Peck, 2008; Sutton & Lambrinos, 2015). The steady increase in urbanization, alongside the popularity of green roofs, suggests a potential key role of green roofs at increasing urban biodiversity if designed correctly (Blaustein et al., 2016). Green roof studies can provide ideal testing grounds for general ecological theory (Vasl & Heim, 2016) being man‐made, and they offer a high level of experimental control. The results of these studies would not only improve theoretical insights but give verified practical tools for green roof designers to implement in their green roof planning and enhance green roof biodiversity. Since green roofs are carefully designed and generally costly, simple manipulations that would stabilize and enhance a diverse plant community—for example, substrate heterogeneity could prove a highly beneficial and a cost‐effective method to increase diversity on green roofs.

Green roof studies have previously targeted the enhancement of species diversity via heterogeneity. Previous studies have manipulated different substrate features (Lundholm, 2009) as well as the mixing of annuals with perennials (Vasl, Shalom, Kadas, & Blaustein, 2017), creating heterogeneous surface features such as logs and pebbles (Walker & Lundholm, 2017) and substrate depth (Heim & Lundholm, 2014).

We established green roof modules and manipulated heterogeneity of a set amount of different substrate components with relatively large subplot size. We predicted that the different substrate niches would support different plant communities which would lead to higher levels of total plant diversity in the more heterogeneous modules. In an attempt to avoid effects caused by specific kinds of heterogeneity (partially mentioned in hypothesis 1), we tested both the commonly manipulated organic components as well as nonorganic components that are commonly used in the green roof industry that have very different features (e.g., weight and water content).

We emphasize that the treatment performed in this study was only the level and type of inner distribution of the total substrate components while total substrate components were kept similar. The goal of this experiment was not to discern the effect that each of the specific treated substrate compositions has on the plant community but instead to isolate the role of substrate heterogeneity on plant diversity. However, following the results of plant communities in control and treated plots, we did analyze plant species distribution within the plot to better our understanding of the processes that took place throughout the experiment.

## 2 METHODS

### 2.1 Experimental design

The experiment included 24 experimental modules that were placed on three school roofs (eight modules per roof) in the city of Haifa, Israel, and monitored for three consecutive growing seasons. Haifa has a typical dry Mediterranean climate with short rainy winters and long, hot, and dry summers. Precipitation events mainly take place between late October and early April. The three schools were “Dinur,” “Ben‐Gurion,” and “Matos” (Table 1). Selected schools were ones with safe access and a suitable roof sealing layer and were relatively near each other.

**Table 1:** Characteristics of the three schools where experiments were placed

School name	Location	Elevation (m asl)	Precipitation (mm)		Average max daily temp. (°C)		
			2014–15	2015–16	Jan 2015	Aug 2015	Jan 2016
“Dinur”	32.79°N, 35.01°E	186	577.4	337.5	19.45	38.19	18.77
“Ben‐Gurion”	32.79°N, 35.00°E	208	585.6	345.5	17.34	34.81	16.94
“Matos”	32.81°N, 34.98°E	264	635.3	347.5	16.37	34.98	16.19

Note

Temperature and precipitation were collected for the second and third years.

Assembly of all experimental modules was completed on 2 December 2013. Prior to the completion of the experimental modules, very few early rains (total of 7 mm over 6 minor rain events) occurred, so the relatively late start should have had little impact on plant development in the first growing season. Module frames (length × width × height: 2,000 × 2,000 × 200 mm) were made of wood and lined with a 0.5 mm waterproof plastic membrane sheet (Wepelen^®^ Aqua Tec, RKW). A 2‐cm deep drainage mat composed of recycled polyethylene foam waste (3RFOAM, “Palziv”) was placed on top of the waterproof plastic membrane sheet. The modules, consistent with green roof practice (FLL, 2008), were placed on a 2° slope on each of the roofs. One drainage point per module was situated 50 mm above the lower‐most corner of the module. A 400 × 400 mm “cushion” made of a coated nonwoven root barrier sheet (Plantex^®^ Gold; DuPont) containing 1 L of large tuff (4–8 mm) was placed on the inner side of the drainage unit to filter runoff water and prevent clogging of drainage. Modules were placed on a synthetic foam sheet (GalFoam – GA400, “Palziv”) to insulate the modules from the roofs and to protect the modules and the roofs from physical damage.

Substrate for all modules was composed of 10% peat, 10% compost, 10% tuff (local volcanic ash—0–8 mm), and 70% processed perlite (imported amorphous volcanic glass—0.6 mm, produced by “Agrical”). Treatments were composed of different levels of dispersion of substrate components. Treatments included the following: (a) homogeneous dispersion (i.e., “HOM”)—all components were homogeneously distributed; (b) mineral heterogeneity (i.e., “M‐HET”)—only mineral components (perlite and tuff) were heterogeneous in their dispersion; (c) organic heterogeneity (i.e., “O‐HET”)—only organic components (compost and peat) were heterogeneous in their dispersion; and (d) mineral and organic heterogeneity (i.e., “M+O‐HET”)—both mineral and organic components were heterogeneous in their dispersion. In order to retain the tuff:perlite and low:high organic matter ratios, the total sum of tuff in this treatment was slightly higher (96.19 L per module) and perlite was slightly lower (479.81 L per module) than other treatments (Table 2). All treatment compositions were achieved by mixing the individual components for a constant period of time in a clean portable electric cement mixer.

**Table 2:** Substrate compositions of the different treatments components. Substrate components quantities (liters and percent) for one module of the different treatments

	Homogeneous		Mineral heterogeneity				Organic heterogeneity				Mineral and organic heterogeneity					
	HOM (1)		M‐HET perlite 1(2a)		M‐HET tuff (2b)		O‐HET high organic (3a)		O‐HET low organic (3b)		M+O‐HET matrix (4a)		M+O‐HET tuff (4b)		M+O‐HET low organic (4c)	
	L	%	L	%	L	%	L	%	L	%	L	%	L	%	L	%
Perlite	504	70	504	80	0	0	430	69	74.8	83.125	405	75	0	0	74.81	83.125
Tuff	72	10	0	0	72	80	61.4	9	10.7	11.875	0	0	85.5	95	10.69	11.875
Compost	72	10	63	10	9	10	69.3	11	2.25	2.5	67.5	12.5	2.25	2.5	2.25	2.5
Peat	72	10	63	10	9	10	69.3	11	2.25	2.5	67.5	12.5	2.25	2.5	2.25	2.5
	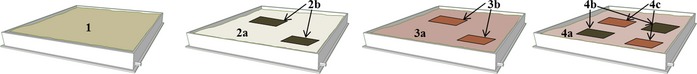															

Note

All modules contained a total of 720 L of substrate but components were dispersed differently within the different treatments. All treated subplots (one treatment for M‐HET and O‐HET and two for M+O‐HET) were separated into two 0.5 × 0.5 m subplots that were placed 250 mm from the module edges.

All modules were subdivided, and four subplots (each subplot: 500 × 500 mm) with plastic frames were positioned in module corners, 250 mm from the module border (Table 2). Subplot plastic frames were placed prior to the filling of the module with the substrate and removed after substrate was filled so that there was no physical barrier between the subplots and the remainder of the module. Diagonal subplots were paired, and each pair consisted 1/8 of the total module area (=0.5 m^2^). In treatments M‐HET and O‐HET, one pair of subplots (randomly chosen) was filled with the additional substrate mix. In treatment M+O‐HET, the two additional substrate mixes were randomly added to the two different pairs. The remaining subplots (in treatments HOM, M‐HET, and O‐HET) were filled with the corresponding volume of the substrate used in the rest of the module.

Seeds of 19 species of local annuals from different families including grasses and nitrogen fixers were collected throughout 2013, and seeds of *Agrostemma githago* (a locally protected species) were purchased from a local wild flower nursery (“Seeds from Zion”) (Table 3). Each of the modules was seeded with a total of 4,000 seeds—200 seeds from each of the 20 species. Seeds were mixed in a bucket with 1 L of sand and evenly distributed over the entire experimental module.

**Table 3:** Species list used in the study alongside their flowering date. Plant species used in the experiment

	Species	Family	Peak flowering	Seed collection date	Collection location
1	*Agrostemma githago* L.	Caryophyllaceae	April–May	–	–
2	*Anthemis pseudocotula* Boiss.	Compositae	March–April	21.8.13	32.71N, 34.95E
3	*Chaetosciadium trichospermum* (L.) Boiss.	Apiaceae	March–April	24.4.13	32.80N, 35.00E
4	*Chrysanthemum coronarium* L.	Compositae	February–April	26.5.13	32.76N, 35.02E
5	*Cichorium endivia* L.	Compositae	April–June	14.11.13	32.76N, 35.02E
6	*Daucus broteri Ten*.	Apiaceae	April–June	2.7.13	32.78N, 34.97E
7	*Echium judaeum* Lacaita	Boraginaceae	March–April	12.6.13	32.78N, 34.97E
8	*Erodium malacoides* (L.) L'Her.	Geraniaceae	January–April	17.3–8.4.13	32.63N, 35.07E
9	*Heliotropium hirsutissimum* Grauer	Boraginaceae	May–October	4–10.8.13	32.76N, 35.02E
10	*Hirschfeldia incana* (L.) Lagr.‐Foss.	Brassicaceae	January–April	12.6.13	32.76N, 35.02E
11	*Lagurus ovatus* L.	Poaceae	March–April	21.8.13	32.71N, 34.94E
12	*Lomelosia prolifera* (L.) Greuter and Burdet	Dipsacaceae	March–May	27.5.13	32.68N, 35.08E
13	*Malva parviflora* L.	Malvaceae	February–April	5.4.13	32.63N, 35.07E
14	*Ricotia lunaria* (L.) DC.	Brassicaceae	January–April	15.3–15.4.13	32.79N, 35.01E
15	*Silene aegyptiaca* (L.) L. f.	Caryophyllaceae	January–April	11–25.3.13	32.63N, 35.07E
16	*Sinapis alba* L.	Brassicaceae	January–April	30.5.13	32.77N, 35.01E
17	*Stipa capensis* Thunb.	Poaceae	March–May	30.4.13	31.58N, 34.94E
18	*Tordylium carmeli* (Labill.) Al‐Eisawi and Juri	Apiaceae	April–June	12.6.13	32.76N, 34.98E
19	*Trifolium purpureum* Loisel.	Fabaceae	March–May	27.5.13	32.64N, 35.06E
20	*Trifolium stellatum* L.	Fabaceae	February–April	5.4.13	32.64N, 35.06E

Note

Annuals are from 10 different families including grasses (Poaceae) and nitrogen fixing legumes (Papilionaceae). All seeds were collected from wild populations except for the locally protected *Agrostemma githago* whose seeds were purchased.

Modules were then covered with a 20 mm layer of medium‐sized (6–20 mm) gravel to avoid wind erosion of perlite‐based substrates and seed scattering before the first rains of the first season.

### 2.2 Plant development measures

#### 2.2.1 Point‐intercept measures

In the beginning of February of 2014, a nondestructive biomass measure was performed once a month throughout the growing seasons using the point‐intercept method (Jonasson, 1988). One hundred metal skewers (diameter of 2.5 mm) were uniformly placed (83.3 mm apart) in each of the modules. Number and identity of green plant organs that intercepted with the skewer were documented. The sum of the yearly touches was used as a biomass proxy, and the identity was used to estimate species distributions within the module. While different growth forms have been shown to have different biomass:intercept ratios, use of this method for repeated monitoring within given experimental units containing several growth forms has been shown to be effective (Bråthen & Hagberg, 2004).

#### 2.2.2 Individual count

At the end of the growing period of each of the species, all dead plants were counted. These data were used to calculate total module yearly Shannon–Wiener diversity index (*H*′).

### 2.3 Subplot level analysis

Point‐intercept data were tracked on the subplot level so that the total sum of intercepts counted in the treated subplots (the two diagonal paired subplots—total of 18 skewers) as well as the respective “control” subplots that contained similar substrate to that in the matrix could be attained for each of the modules.

A sum of yearly species identity for each of the potential treated and “control” subplots was calculated. The seven different subplots included were—one subplot value for treatment HOM, and two for each of the other three treatments—M‐HET (tuff and perlite subplots), O‐HET (low and high organic subplots), and the M+O‐HET (tuff and low organic subplots). The point‐intercept subplot communities were used to calculate Bray–Curtis distances for all three growing seasons.

### 2.4 Substrate change monitoring

Core samples (50 ml) were collected from each module at the end of each growing season after substrate was dry (18 September 2014, 20 September 2015, and 8 July 2016) to determine whether substrate composition differences were maintained over time. Two paired samples were taken from both the matrix and subplot at distance of 100 mm from either side of the initial subplot border with the module matrix. In light of the substantial weight differences between tuff and perlite, samples were initially weighed to assess changes in tuff:perlite ratios over time and then burned for 12 hr at 550°C at the Neve Ya'ar Agricultural Center to obtain percent organic matter. Since percent organic matter is a weight factor and the original substrate mixes were by volume, we could not compare percent organic matter when the two samples differed in their tuff:perlite ratio as their weight differences mask organic matter differences. For this reason, we could only use percent organic matter results for treatments HOM and O‐HET. Substrate moisture (volumetric water content) was measured once a month throughout the growing seasons of the years of the study, with an ECH_2_O EC‐5 frequency domain probe (Decagon Devices Inc.). Measurements were taken on either side (distance of 250 mm) of the initial subplot border with the module matrix. We used only the January measurements that represent the peak of the rainy season.

### 2.5 Statistical analysis

The experiment consisted of four different treatments, with six replicated modules equally distributed in three blocks, that is, two samples of each treatment on each of the three schools. Repeated measures one‐way ANOVA (SPSS 23; SPSS Inc.) was performed for total plot point‐intercept biomass proxy and Shannon–Wiener diversity index as well as for assessing the differences between the subplot and the module matrix (weight, moisture, and percent organic matter) throughout the 3 years of the experiment. Parametric assumptions including homogeneity of variance (Levene's test) and normal distribution (Shapiro–Wilk test) of residuals were tested .

Data were transformed (specific transformations are reported at each relevant test) when parametric assumptions were not met. Greenhouse–Geisser corrections for degrees of freedom were used when sphericity assumptions were not met.

Community dissimilarity between modules and between subplots was calculated using Bray–Curtis differences. The data were visualized in nonmetric dimensional scaling plots (NMDS), using the meta‐DATA function in the vegan package of R (Oksanen et al., 2013). A nonparametric multivariate analysis of variance PERMANOVA on Bray–Curtis dissimilarities with 999 permutations was performed on whole module species abundance data and subplot community point‐intercept data for each of the years using “adonis” function of “vegan” package in R, with block, treatment, and their interactions as predictors. Since PERMANOVA tests do not have post hoc procedures, when treatment was statistically significant, we performed pairwise *t* tests on each of the combinations to establish which were different. Critical *p*‐values were corrected following the “Benjamini–Hochberg” false discovery correction (Benjamini & Hochberg, 1995).

## 3 RESULTS

### 3.1 Total module results

**Figure 1:**
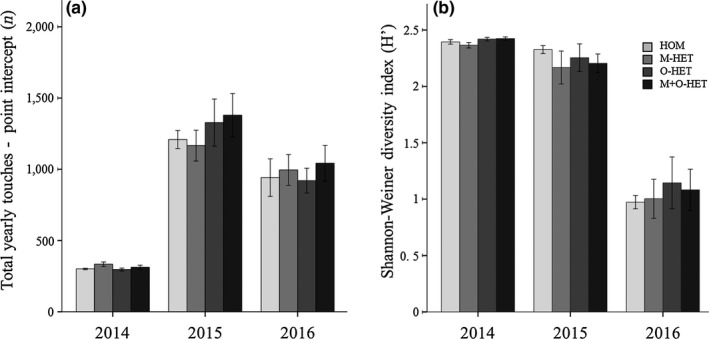
Treatment effect on plant community biomass proxy and diversity values over the 3 years of the experiment. Biomass proxies obtained from total yearly point‐intercept data (a) showed no treatments effect on any of the years. Plant community diversity index (H′) showed a decrease over the years but no treatment effect (b). The four different treatments included—homogeneous substrate (HOM), heterogeneity of mineral components (M‐HET), heterogeneity of organic component (O‐HET), and heterogeneity of mineral and organic heterogeneity (M+O‐HET). Error bars in graphs represent ± 1 *SE*

Biomass proxy (point intercept) did not change with substrate heterogeneity, but increased from the first to the second year and decreased in the third year (Figure 1a) (repeated measures one‐way ANOVA, *p* < .001; Table 4).

**Table 4:** Repeated measures ANOVA table for treatment effects on plant community biomass proxy and diversity values

	Source of variance	Point intercept			Shannon–Wiener		
		*df*	*F*	*p*	*df*	*F*	*p*
Between subject	Treatment	3,20	0.55	0.66	3,20	0.37	0.78
Within subjects	Year	1.54,30.76	95.1	**<0.001**	2,40	237.68	**<0.001**
	Year*Treatment	4.61,30.76	0.4	0.94	6,40	0.51	0.8

Note

Repeated measures one‐way ANOVA on the effects on point intercept and Shannon–Wiener diversity index in the experimental modules over the 3 years of the experiment. Degrees of freedom were adjusted based on Greenhouse–Geisser adjustments. Significant results appear in bold.

Shannon–Wiener diversity index (*H*′) did not change with substrate heterogeneity either, but decreased over the 3 years of the experiment (Figure 1b) (repeated measures one‐way ANOVA (*x* ^2^‐transformed), *p* < .001; Table 4).

**Figure 2:**
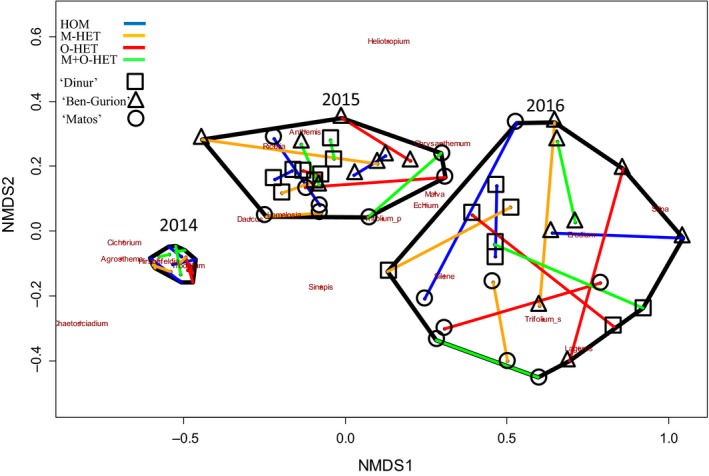
Nonmetric dimensional scaling (NMDS) plotting of similarity of the community compositions in the different modules over the 3 years of the experiment, grouped by treatment and school. Nonmetric dimensional scaling was calculated for all modules of all years. Modules from each year are enclosed in separate black polygons. Each two modules of the same treatment on the same school are connected with a line. Each year's plots are enclosed within a black polygon. Colors represent treatment and shapes represent school identity

Plant community similarities displayed in nonmetric dimensional scaling (NMDS) in Figure 2 depict the small effect of substrate heterogeneity as opposed to the change and divergence depicted over time as well as the strong effect of school identity.

Bray–Curtis distances of whole module communities for each of the years showed a significant treatment effect only on the first year (PERMANOVA, *p* = .01; Table 5) while school block effects were significant on years 1 and 3 (*p* < .001 and *p* < .01, respectively; Table 5). Pairwise comparisons performed on the first‐year results found that only treatments M‐HET and O‐HET had a significant treatment effect between them (Pseudo‐*F*(1) = 3.03, *p* = .004).

**Table 5:** PERMANOVA table for treatment and block effects on year module community assemblages for each year

	*df*	2014		2015		2016	
		Pseudo‐*F*	*p*	Pseudo‐*F*	*p*	Pseudo‐*F*	*p*
Treatment	3	2.03	**0.01**	0.91	0.52	0.21	0.99
Block	2	4.86	**<0.001**	1.84	0.09	4.35	**0.005**
Treatment*block	6	0.83	0.75	0.9	0.57	0.62	0.85

Note

PERMANOVA results per year performed on the Bray–Curtis distances between the community assemblages in the different modules with treatment, block (school), and their interaction used as explanatory variables. Significant results appear in bold.

### 3.2 Subplot level analysis

**Figure 3:**
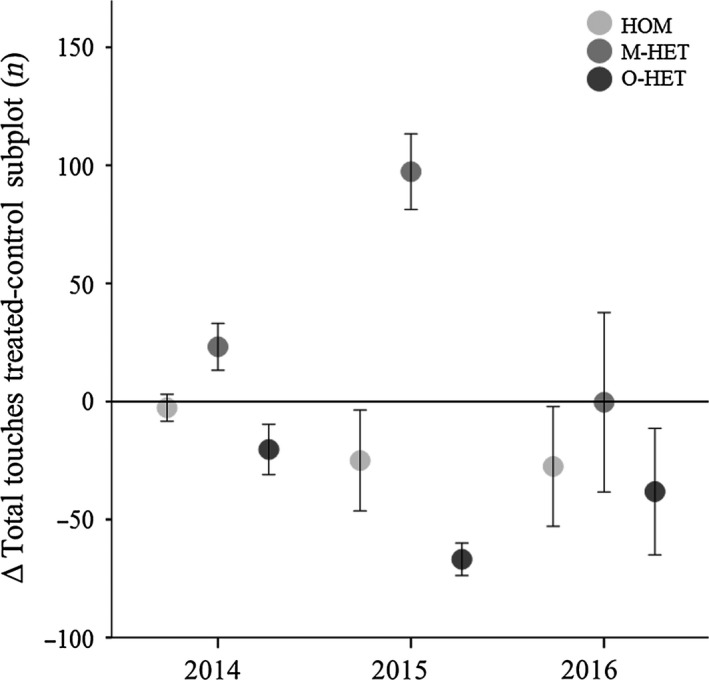
Differences in a biomass proxy between treated and control subplots over the 3 years of the experiment. Mean differences displayed of the treated and control subplots in the homogeneous (HOM), mineral heterogeneity (M‐HET), and organic heterogeneity (O‐HET) treatments for all 3 years. Repeated measures ANOVA showed a treatment effect between treatment M‐HET and the HOM and O‐HET treatments but showed no year effect

Biomass proxy differences between the sums of the two treated and the two control subplots showed a significant effect for treatment (Repeated measures one‐way ANOVA, *F*_2,15_ = 16.84, *p* < .001) while year (*F*_1.23,18.38_ = 1.11, *p* = .32) and year*treatment interaction (*F*_1.23,18.38_ = 2.45, *p* = .07) were not significant (Figure 3). Post hoc tests (Tukey's HSD) showed that the differences between control and treated subplots in treatment M‐HET were higher than those in treatments HOM and O‐HET during the first 2 years.

Bray–Curtis distances of the seven subplot communities (HOM, M‐HET‐tuff, M‐HET‐perlite, O‐HET‐low, O‐HET‐high, M+O‐HET‐tuff, and M+O‐HET‐low) for each of the years (based on point‐intercept data) showed a significant treatment effect only on the first year (PERMANOVA, *p* < .001; Table 6). School (=block) effects were significant on all 3 years (*p* = .02, *p* = .02, and *p* < .001 respectively; Table 6). Pairwise comparisons performed on the first year's results (Table 7) showed that the communities present in the tuff subplots of M‐HET were significantly different from all other communities excluding the communities on the tuff subplots in M+O‐HET. The communities in low organic subplots in O‐HET were also significantly different from all other communities excluding the communities on low organic subplots in M+O‐HET and the high organic communities in O‐HET subplots.

**Table 6:** PERMANOVA for treatment and block effects on yearly subplot community assemblages

	*df*	2014		2015		2016	
		Pseudo‐*F*	*p*	Pseudo‐*F*	*p*	Pseudo‐*F*	*p*
Treatment	6	2.34	**<0.001**	1.27	0.17	1.1	0.36
Block	2	2.07	**0.02**	2.15	**0.02**	8.63	**<0.001**
Treatment*block	12	0.91	0.69	0.69	0.91	0.48	0.99

Note

PERMANOVA results per year performed on the Bray–Curtis distances between the community assemblages in the seven different subplots (HOM, tuff, and perlite in M‐HET, low and high organic in O‐HET, and tuff and low organic in M+O‐HET) with treatment, block (school), and their interaction used as explanatory variables. Significant results appear in bold.

**Table 7:** Pairwise PERMANOVA tests for the subplot community assemblages of 2014

	HOM	M‐HET tuff	M‐HET perlite	O‐HET low organic	O‐HET high organic	M+O‐HET tuff
M‐HET tuff	**0.005**					
M‐HET perlite	0.774	**0.007**				
O‐HET low organic	**0.008**	**0.002**	**0.01**			
O‐HET high organic	0.247	**0.004**	0.4	**0.032**		
M+O‐HET tuff	0.289	**0.037**	0.763	**0.016**	0.296	
M+O‐HET low organic	0.173	**0.004**	0.284	0.653	0.582	0.532

Note

*p*‐Value results for pairwise PERMANOVA tests of the different subplot communities for the 2014 season (obtained from yearly point‐intercept data). Test results show that treatment M‐HET tuff subplots differs from all other subplot communities and O‐HET low organic subplots differs from all other subplot communities with the exception of M+O‐HET low organic. *p*‐Values <0.05 appear in bold, and results significant after the “Benjamini–Hochberg” correction appear with a gray background.

**Figure 4:**
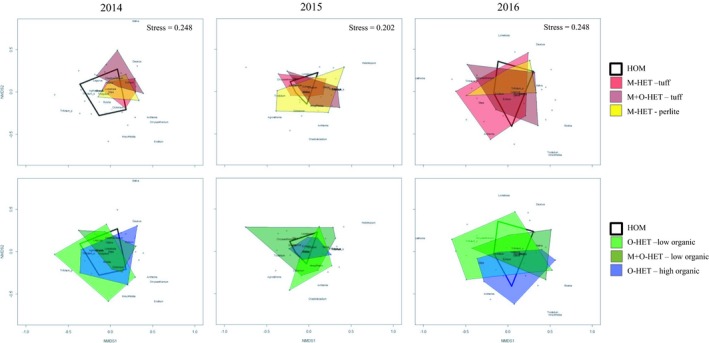
Nonmetric dimensional scaling (NMDS) plotting of similarity of the community compositions in the different subplots over the 3 years of the experiment, grouped by treatment. Nonmetric dimensional scaling for all seven subplot types (performed for each year separately). Communities are surrounded by a polygon. For visual aid, each plot is depicted twice—once on the top row and once on the bottom row. On the top row, polygons of the homogeneous treatment as well as the subplots of the three different mineral heterogeneity subplots are displayed. On the bottom row, polygons of the homogeneous treatment and the three different organic heterogeneity subplots are displayed

Nonmetric multidimensional scaling (NMDS) visualization of plant communities (based on point‐intercept data) in the seven different subplots (Figure 4) depicts the differences between plant communities in subplots over time.

### 3.3 Substrate differences over time

**Figure 5:**
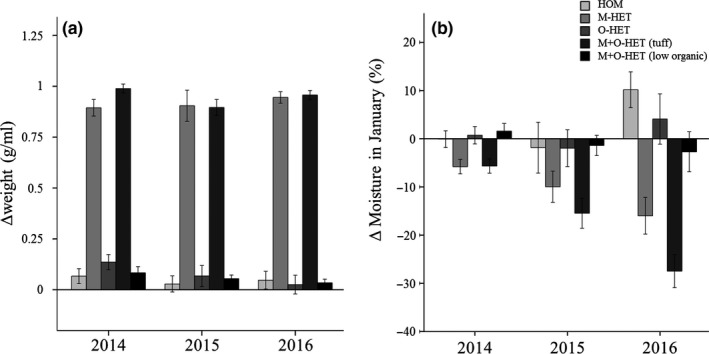
Differences in substrate core sample weight and January substrate moisture between treated subplots and their surroundings over the 3 years of the experiment. Differences in weight (a) and January moisture (b) between treated subplots and their surrounding substrate. Post hoc tests showed that for weight and January moisture measures, M‐HET (tuff) and M+O‐HET (tuff) differences were significantly different from the other subplot differences

Differences between core sample weights for each of the treatments (treated subplot as well as the substrate near it) (Figure 5a) found a treatment effect (repeated measures one‐way ANOVA, *p* < .001; Table 8). Tukey's post hoc tests showed that the tuff subplots in treatments M‐HET and M+O‐HET were significantly heavier than the HOM and low organic subplots from treatments O‐HET and M+O‐HET.

**Table 8:** Repeated measures ANOVA table for treatment effects on the substrate weight, January moisture and percent organic matter differences between treated and control subplots

	Source of variance	Weight			Moisture			Organic matter		
		*df*	*F*	*p*	*df*	*F*	*p*	*df*	*F*	*p*
Between subject	Treatment	4,25	322.45	**<0.001**	4,25	16.06	**<0.001**	1,10	23.4	**0.001**
Within subjects	Year	2,50	1.98	0.15	2,50	1.14	0.33	2,20	1.13	0.34
	Year'Treatment	8,50	0.87	0.55	8,50	1.23	0.3	2,20	3.04	0.07

Note

Repeated measures ANOVA for effects on differences between treated and control subplot substrate weight, moisture in January and percent organic matter over the 3 years of the experiment. Significant results appear in bold.

A treatment effect (*p* < .001; Table 8) was found for differences in January moisture measurements (repeated measures one‐way ANOVA arcsin‐square root‐transformed) (Figure 5b). Post hoc tests showed that the moisture differences for the two tuff subplots were significantly drier while other subplots were not.

**Figure 6:**
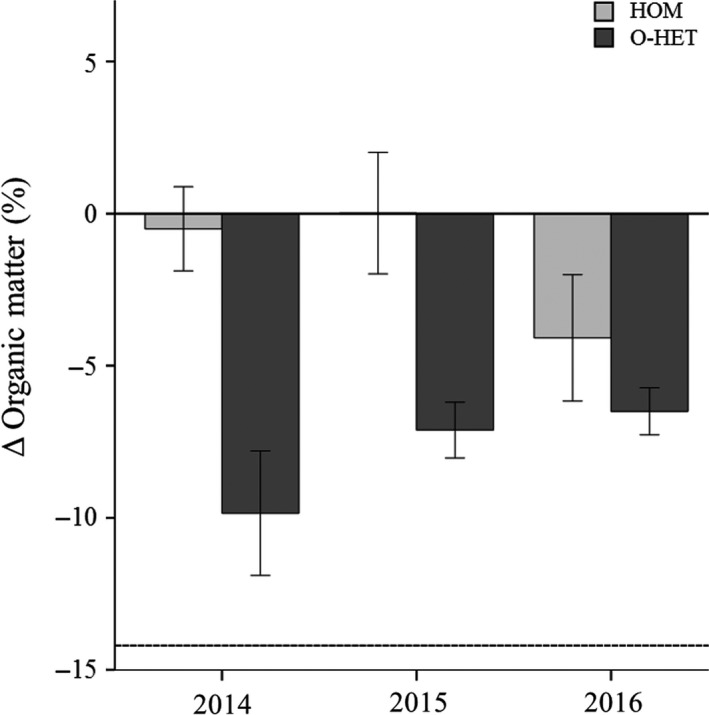
Differences in substrate core sample percent organic matter between homogeneous and low organic subplots and their surroundings over the 3 years of the experiment. The difference in percent organic matter between homogeneous (HOM) and low organic (O‐HET) subplots and their surroundings. Core samples were taken at the end of each growing season. Dotted line marks the difference in the initial levels of the low organic substrate and its surroundings

Differences between percent organic matter (arcsin‐square root‐transformed) of treatments HOM and O‐HET showed that there was a statistically significant treatment effect (Repeated measures one‐way ANOVA, *p* = .001) while differences were larger in O‐HET subplots and that year and year*treatment interaction were not significant (Table 8, Figure 6).

## 4 DISCUSSION

In our experiment, we did not find a positive effect of substrate heterogeneity on plant diversity throughout the 3 years of the experiment. The inner module documentation of plant specimen locations suggested that plants were locally affected by substrate treatments but only on the first year. Finally, substrate yearly changes were documented and suggest that differences between substrate treatments were maintained over the years.

As portrayed above, while positive HDR is a generally accepted phenomenon with strong theoretical backing, soil HDR (especially in studies comparing similar sized units) is often not positive (Stein & Kreft, 2015).

Since experimental soil studies are typically limited in size, the size of the modules used in the experiments was targeted as the potential cause to this discrepancy (Walker & Lundholm, 2017). The even smaller patches (i.e., subplot) within the experimental modules may not be large enough to sustain individuals of a different species. Presumably, if only soil experimental studies were larger in size, the patches within the experimental modules could sustain individuals from different species and the studies would show a positive HDR in accordance with general HDR findings.

A meta‐analysis performed entirely on soil manipulation studies (Tamme et al., 2010) strengthened this assumption and claimed that experimental studies’ negative relationship was limited by size of experimental units. The meta‐analysis contained several large‐scaled presumably “experimental” studies that showed a positive HDR which allowed the researchers to reach this conclusion. However, the terminology used in this study may have been misleading, as they define “experimental” studies as binary studies with homogeneous and heterogeneous areas/modules being compared and not as commonly defined experimentally manipulated studies. As a matter of fact, the vast majority of the studies included were confounded in their size (module < 0.25 m^2^) (Tamme et al., 2010) and it was not possible to successfully isolate the targeted size factor.

An additional inherent problem with most soil heterogeneity studies conducted in the past was that they were often limited to only one growing season (Gazol et al., 2013; Price, Gazol, Tamme, Hiiesalu, & Pärtel, 2014; Tamme et al., 2016) and therefore did not allow the testing of community‐level processes such as dispersal.

Our study had large enough subplots to sustain several individuals of a certain species, involved both mineral and organic substrate manipulations and lasted more than one growing season. However, we did not find a positive HDR or a positive effect on biomass in experimental modules. Interestingly, another large‐scaled ground‐level 15‐year experimental soil study (Baer, Blair, & Collins, 2015) that was published after Tamme et al. (2010) did not find a positive HDR either.

We believe that the theoretical considerations regarding the effect of subplot size on local populations' persistence within the subplots (Kadmon & Allouche, 2007) support a good understanding of this system. Our findings showed that community composition in treated modules of O‐HET and M‐HET as well as the community compositions inside the treated subplots in these treatments did differ during the first year. Substrate mineral and organic differences were maintained throughout the duration of the experiment while module and subplot communities no longer responded to these differences after the first year. These findings allow us to point toward a potential effect on the community level that was not previously explicitly examined in experimental studies.

In response to the three potential hypotheses presented in the introduction:

1. Realism in the method of creation of heterogeneity

On green roofs, as opposed to ground‐level experiments, substrates that are used are intrinsically human‐made; therefore, these manipulations are representative of the dynamics that are commonly predicted on green roofs. In addition, the organic manipulations that were especially targeted as nonrealistic showed similar behavior to the mineral treatment.

- Subplot size effect on individuals

The initial first‐year response of community composition and subplot biomass response over all 3 years imply that individuals in subplots were affected by the subplots' unique substrate compositions and that subplot size was sufficient for the maintenance of individuals from different species.

- Subplot size effect on populations

Finally, the change in response to the community composition over time may imply that population and community dynamics might be playing a role at structuring the communities in these experimental modules. Subplot communities in the second and third seasons may have been altered by a “mass effect” (Shmida & Ellner, 1984) of propagules from its surroundings while losing many of the propagules of their locally “adapted” community to the unfavorable surroundings.

The lack of significance between treatment M+O‐HET matrix and subplots may result from the reduction in the area surrounding the subplots. The excessive fragmentation into many units may have prevented the establishment of any community that would be subplot‐specific (Kadmon & Allouche, 2007). This may also imply that positive HDR is limited by abiotic heterogeneity.

Our findings also suggest a relatively strong effect of block location within the city on plant community development. This could result from different microclimates in the different parts of the city (mainly wind exposure and specific rain events at the end of the season that could affect plant development) as well as the specific site characteristics (height of roof and distance to potential pollinating insect communities) as was displayed in previous studies (Braaker, Ghazoul, Obrist, & Moretti, 2014). These differences in plant communities in identically designed modules should be considered in the future design of diverse green roofs within the city. Our study suggests that the specific location of the roof could eventually support a different plant community structure and so increase the total green roof beta diversity.

Finally, in order to properly examine the viability of distinct plant populations within the different niches, we suggest the construction of experimental designs that would manipulate both module and subplot sizes. This experimental design would include subplots that could potentially support a distinct population over time. This would allow the point to be addressed appropriately. Alternatively, seed dispersal between subplots and the matrix could be directly manipulated to test for the same effect. These studies would preferably also include heterogeneity in non‐nutrient substrate components which is very rarely manipulated.

## CONFLICT OF INTEREST

All authors declare they have no conflict of interest.

## AUTHORS CONTRIBUTION

AV, GJK, and LB conceived the ideas and designed methodology; AV and BYS collected the data; AV and BYS analyzed the data; AV led the writing of the manuscript. All authors contributed critically to the drafts and gave final approval for publication.

## Data Availability

Sampling data and R script: https://doi.org/10.5061/dryad.86qh1tm.
